# Two cases of oligometastatic castration‐resistant prostate cancer detected by diffusion‐weighted whole‐body imaging with background body signal suppression

**DOI:** 10.1002/iju5.12146

**Published:** 2020-02-17

**Authors:** Yuki Shimizu, Nobuyuki Nakajima, Kentaro Nagao, Masahiro Nitta, Masanori Hasegawa, Yoshiaki Kawamura, Toshiki Kazama, Sunao Shoji, Taro Takahara, Akira Miyajima

**Affiliations:** ^1^ Department of Urology Tokai University School of Medicine Isehara Japan; ^2^ Department of Diagnostic Radiology Tokai University School of Medicine Isehara Japan; ^3^ Department of Biological Engineering Tokai University School of Biological Engineering Isehara Japan

**Keywords:** CRPC, DWIBS, metastasis‐directed therapy, oligometastasis, prostate cancer

## Abstract

**Introduction:**

Treatment for oligometastasis in prostate cancer has changed from systemic therapy to metastatic lesion‐targeted therapy. Early detection of metastatic lesions and assessment of the treatment response have become very important. Therefore, we started to perfume assessments with whole‐body magnetic resonance imaging, especially diffusion‐weighted imaging with background body signal suppression, as a modality to detect metastasis in patients with prostate cancer.

**Case presentation:**

We encountered two cases of castration‐resistant prostate cancer in which oligometastasis was detected by diffusion‐weighted imaging with background body signal suppression. Metastasis‐directed therapy was initiated for to treat the lesions in each case. The treatment was effective for disease control and symptom relief. Diffusion‐weighted imaging with background body signal suppression could detect new lesions at an early phase and delineate changes in lesions immediately after therapy.

**Conclusion:**

Diffusion‐weighted imaging with background body signal suppression enables early decision‐making for metastasis‐directed therapy compared with conventional imaging modalities. Further, metastasis‐directed therapy targeting oligometastatic lesions detected by diffusion‐weighted imaging with background body signal suppression may improve patients’ overall survival and quality of life.

**Abbreviations & Acronyms**
ADT: androgen deprivation therapy
BCR: biochemical recurrence
BS: bone scintigraphy
CRPC: castration‐resistant prostate cancer
CT: computed tomography
DWI: diffusion‐weighted image
DWIBS: diffusion‐weighted imaging with background body signal suppression
EBRT: external beam radiotherapy
MRI: magnetic resonance imaging
PSA: prostate‐specific antigen
RP: radical prostatectomy
SBRT: stereotactic body radiotherapy
SRT: salvage radiation therapy

**Keynote message**

We encountered two cases of CRPC in which oligometastasis was detected by DWIBS.

## Introduction

There has been a paradigm shift in prostate cancer treatment owing to the development of new targeted drugs, the implementation of robot‐assisted surgery, and use of diagnostic imaging. Although we have treated prostate cancer patients with metastasis using systemic therapy, local radiation therapy for oligometastasis has been selected recently.^1^ The therapy has been suggested to be effective at treating prostate cancer patients with oligometastasis.^2^, ^3^, ^4^, ^5^ The European Organisation for Research and Treatment of Cancer Imaging Group recommends imaging at a PSA level of 0.2–1.0 ng/mL to exclude multiple metastases and detect oligometastasis for in cases of BCR after RP.^6^ Therefore, diagnostic imaging in prostate cancer has become extremely important. Since 2016, we started using whole‐body MRI, especially DWIBS, as a modality to detect metastasis in prostate cancer.

**Figure 1:**
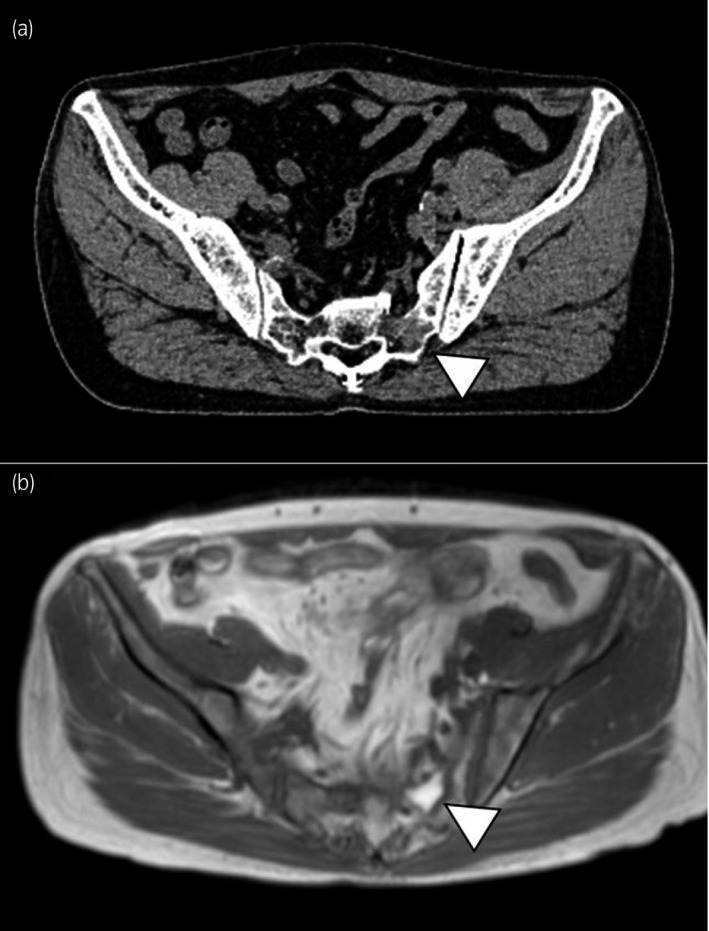
A 75‐year‐old man with BCR after RP in 2008 and SRT in 2013. In 2017, his PSA level increased to 0.44 ng/mL. Osteolytic bone metastasis in the sacrum cannot be detected with CT (a), without the results of DWIBS (b).

DWIBS is a whole‐body MRI method first reported by Takahara *et al*. in 2004.^7^ DWI is an imaging technique based on images that are created through the analysis of the random movement of water at a molecular level. Highly cellular tissues such as tumor tissues suppress water diffusion, creating a strong signal. DWI can show cancer activity and detect new lesions in almost real time. The advantage of DWIBS is the strong contrast of positive signals against normal tissues; thus, DWIBS is useful for identifying primary and metastatic lesions. DWIBS can also detect osteolytic changes, lymph node metastasis, and visceral metastasis in addition to osteoblastic changes.^8^, ^9^, ^10^ We encountered cases of CRPC wherein we could not detect osteolytic bone metastasis in the sacrum with CT (Fig. 1a), without the results of DWIBS (Fig. 1b).

Herein, we describe two CRPC patients who underwent metastasis‐directed therapy on the basis of DWIBS findings.

## Case presentation

### Case 1

**Figure 2:**
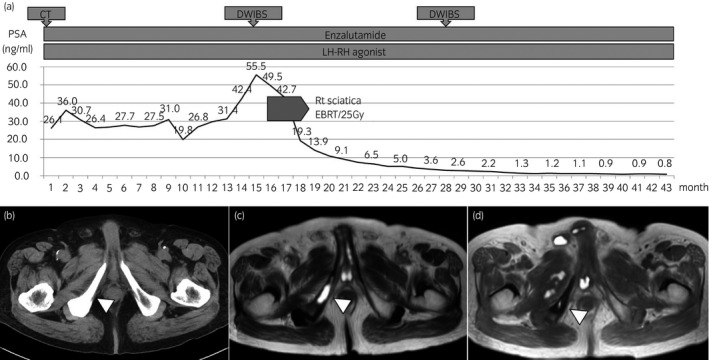
Case 1. A 74‐year‐old man with CRPC after RP and SRT (a). Enzalutamide administration is initiated. Sclerosis of right sciatica is detected with CT. (b) After the administration of enzalutamide, his PSA level initially remains at 20–30 ng/mL, but it increase within 1 year of enzalutamide treatment. Subsequently, DWIBS shows right sciatic metastasis (c). After 1 year of EBRT for right sciatic metastasis, the follow‐up DWIBS study shows intensity reduction in the lesion (d).

A 74‐year‐old man underwent RP in 2003, and the pathology report showed GS3 + 4 adenocarcinoma, pT2c without capsular invasion, and a positive surgical margin (Fig. 2a). BCR was found at 2 years after RP, and SRT was initiated at the prostatic floor (66 Gy). At 5 years after SRT, his PSA level began to increase again so ADT was initiated. We determined that the lesion was castration‐resistant and initiated enzalutamide administration. Then, the PSA levels were 26.1 ng/mL and sclerosis of the right sciatica was detected with CT (Fig. 2b). After the administration of enzalutamide, the PSA level remained at 20–30 ng/mL, but it began to increase within 1 year. Subsequently, he underwent DWIBS, which indicated only right sciatic metastasis (Fig. 2c), and that lesion was radiated with EBRT (25 Gy). While the patient was treated with EBRT, enzalutamide administration was continued because the PSA level remained low (0.98 ng/mL) for over 2 years after EBRT. The follow‐up DWIBS study showed intensity reduction in that lesion (Fig. 2d).

### Case 2

**Figure 3:**
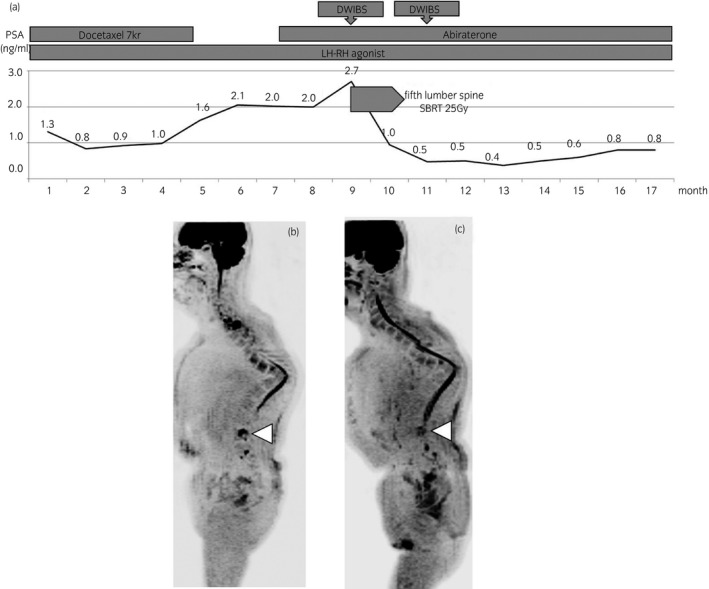
Case 2. A 79‐year‐old man with CRPC after EBRT of the whole pelvis (50 Gy) and prostate (66 Gy) (a). At 2 years after EBRT, his PSA level increases again, and ADT was initiated. In 2016, the PSA levels began to rise again and abiraterone therapy is induced after docetaxel therapy. His PSA level continues to show an increasing trend, from 1.6 to 2.7 ng/mL. Subsequently, we performed DWIBS and show the metastatic lesion in the fifth lumbar spine (b). After 1 month of SBRT for the metastatic lesion, the follow‐up DWIBS study shows intensity reduction in the lesion (c).

A 79‐year‐old man had a PSA level of 228 ng/mL in 2010 and underwent a prostate biopsy, which identified GS5 + 4 adenocarcinoma. A metastatic lesion was not detectable by BS and CT at this time. We then performed EBRT of the whole pelvis (50 Gy) and prostate (66 Gy). At 2 years after EBRT, his PSA level began to increase so ADT was initiated. In 2016, the PSA level began to rise again and docetaxel therapy was started. However, the therapy could not be continued because of myelosuppression. Following this, abiraterone administration was started (Fig. 3a); the PSA level continued to increase from 1.6 to 2.7 ng/mL. Subsequently, we performed DWIBS, which detected a metastatic lesion in the fifth lumbar spine (Fig. 3b). The lesion was radiated by SBRT (25 Gy/3 fraction). At 1 month after SBRT, his PSA level decreased to 1.0 ng/mL, and DWIBS showed intensity reduction in the lesion (Fig. 3c). At 4 months after SBRT, his PSA level remained low (0.37 ng/mL).

## Discussion

The detectability of bone and lymph node metastasis with whole‐body MRI is superior to that with CT, and whole‐body MRI can be an alternative modality to BS and CT in staging diagnosis.^10^ Whole‐body MRI has several merits that include the ability to perform repeated tests over time, cost‐effectiveness in our country, no radiation exposure, no use of contrast material, and comprehensiveness with one examination for bone, other organs, and lymph nodes. Especially, the anti‐tumor effects of therapy have to be examined at several time points during treatment. In addition, whole‐body MRI can delineate not only osteoblastic changes but also osteolytic changes, and whole‐body MRI appears to be superior to BS with regard to this point. Taken together, DWIBS reduces the various burdens on patients.

In contrast, the limitation of DWIBS is that it is difficult to detect small lung metastasis because the lung contains air and its size fluctuates with breathing. Air and implanted metal are causes of artifacts in diffusion‐weighted imaging. DWIBS has another disadvantage for use in daily medical care. Physicians cannot provide feedback to patients immediately, because false‐positive lesions such as lesions in the intestinal tract, brain, and spleen must be excluded. Another controversial point is whether DWIBS can detect lesions early. A combination of whole‐body MRI and multi‐parametric prostate MRI detected metastatic lesions or local recurrence in 16/76 (21%) of BCR cases after RP in patients with a median PSA level of 0.36 ng/mL.^11^

DWIBS can detect new lesions with a slight change in the PSA level in CRPC and delineate reduction in the lesion size immediately after local treatment. Additionally, evaluation by DWIBS can be scheduled repeatedly. Therefore, DWIBS enables early decision‐making for metastasis‐directed therapy compared with conventional imaging modalities. Particularly in case 1, we were able to focus on the treatment of the oligometastatic lesion in sciatica, and we did not have to change the systemic therapy from enzalutamide.

## Conclusion

In our cases, DWIBS demonstrated that it can detect oligometastasis and aid in early decision‐making to treat CRPC patients because it reflects the real‐time disease status. This finding suggests that the use of DWIBS may prolong overall survival and improve patients’ quality of life by early treatment intervention such as metastasis‐directed therapy that targets oligometastatic lesions.

## Conflict of interest

The authors declare no conflict of interest.
